# Paramagnetic and Luminescent Properties of Gd(III)/Eu(III) Ascorbate Coordination Polymers

**DOI:** 10.3390/molecules30132689

**Published:** 2025-06-21

**Authors:** Marco Ricci, Fabio Carniato

**Affiliations:** Dipartimento di Scienze e Innovazione Tecnologica, Università del Piemonte Orientale, Viale T. Michel 11, 15121 Alessandria, Italy; marco.ricci@uniupo.it

**Keywords:** coordination polymers, ascorbate, nanoparticles, paramagnetic probes, luminescent properties

## Abstract

Gadolinium-based contrast agents (GBCAs) are the gold standard as MRI probes but are nowadays facing medical limitations and environmental concerns. To address these issues, novel strategies focus on the optimization of Gd(III)-based probes. One promising approach involves incorporating Gd(III) into nanoparticles, particularly coordination polymers, which offer improved relaxivity. In this study, we explore the self-assembly of Gd(III) ions with ascorbate ligand, forming extended coordination polymer architectures. Our investigation focuses on understanding the impact of nanoparticles’ growth and aggregation on their relaxivity properties. Notably, the controlled aggregation process leads to a different distribution of the Gd(III) in the surface and in the bulk of the nanoparticles, mainly responsible for their longitudinal relaxivity. Additionally, the introduction of Eu(III) into the network enables the development of a dual-modal probe with paramagnetic and optical features.

## 1. Introduction

Magnetic Resonance Imaging (MRI) is a very promising diagnostic technique, offering a non-invasive and high-resolution visualization of soft tissues. The quality of the images in terms of contrast is significantly enhanced by the administration in the patients of contrast agents (CAs), able to reduce the relaxation times of water protons in biological tissues [1,2]. Gadolinium-based contrast agents (GBCAs) remain the gold standard in clinical practice as *T*_1_ MRI probes due to their paramagnetic properties [2,3]. However, clinically used low molecular weight Gd(III) chelates show intrinsic limitations, including low longitudinal relaxivity values at clinical magnetic field (>0.5 T), rapid renal clearance, and concerns regarding long-term tissue deposition and toxicity [4,5,6,7]. Moreover, the increasing use of Gd(III)-based contrast agents has led to environmental impact due to their persistence in water sources [8,9,10,11,12]. Additionally, while these agents are widely employed in clinical practices, the availability of Gd(III) is increasingly impacted by geopolitical and economic factors, as rare earth elements are not uniformly distributed worldwide. As a result of these obstacles, significant research efforts have been directed toward the development of novel alternatives. Two principal strategies have emerged in the last decade: the replacement of Gd(III) with other paramagnetic metals that offer greater biocompatibility and cost-effectiveness [13,14,15,16] or the optimization of Gd(III)-based probes to maximize their relaxometric efficiency and minimize environmental concerns [17,18]. These issues have driven extensive research toward the development of novel Gd(III)-based materials with enhanced relaxivity, improved stability, and optimized pharmacokinetic properties. One promising approach is the use of nanoparticles as carriers for contrast agents, offering several advantages over conventional low molecular weight chelates [17]. Nanoparticles can encapsulate a high payload of paramagnetic centers, reducing renal clearance and allowing for prolonged circulation times, which enhances MRI signal intensity. The surface of nanoparticles can be functionalized to improve biocompatibility, target specific tissues, or modulate interactions with biological media, enhancing contrast efficiency. This engineering also enables the development of multimodal or theranostic systems, combining imaging and therapeutic functionalities [19]. Various nanosystems, including liposomes [20], dendrimers [21,22], polymeric micelles [23,24], nanogels [25,26,27,28,29,30], silica nanoparticles [31,32], and coordination polymers [33,34,35], have been explored as contrast agents. These architectures proved to be a viable option to improve contrast efficacy (relaxivity) by reducing molecular tumbling in solution, which enhances the correlation time for water proton relaxation, leading to more efficient MRI contrast at clinical magnetic fields. However, optimizing relaxivity requires careful consideration of additional factors. Specifically, the hydration state of the metal center, the average residence lifetime of water molecules, and the longitudinal and transverse electronic relaxation times of the metal ion must be controlled during probe design. Recently, coordination polymers have garnered increased interest in this field. For instance, nanoparticles based on Gd(III)-ascorbate were proposed as novel MRI nanoprobes [36]. These samples were found to be stable in aqueous suspension and exhibit higher relaxivity compared to clinically used contrast agents. Interestingly, during the synthesis, a gradual increase in the nanoparticle dimensions was observed, accompanied by variations in the recorded relaxivity values.

An important novel aspect of this work is the investigation of how the aggregation state of Gd(III)-ascorbate coordination polymers (Gd-Asc) influences their relaxometric properties. Morphological and chemical analyses were carried out over time, during the particle formation process. ^1^H Nuclear Magnetic Relaxation Dispersion (NMRD) profiles, recorded across a broad range of magnetic fields and temperatures, were also obtained, which confirmed the correlation between aggregation state and relaxometric properties. A second novel element of our work is the incorporation of a luminescent lanthanide ion into the inorganic framework to alter the intrinsic emission properties of the system. For this goal, we explored the possibility to confine Eu(III) ions in the Gd(III)-ascorbate nanoparticles in order to confer both paramagnetic and luminescent properties. This study offers valuable insights into the mechanisms underlying the performance of these systems, emphasizing the critical role of controlling the synthetic process to optimize their relaxometric and optical properties.

## 2. Results and Discussion

### 2.1. Nanoparticles Based on Gd(III)-Ascorbate (Gd-Asc)

**Figure 1:**
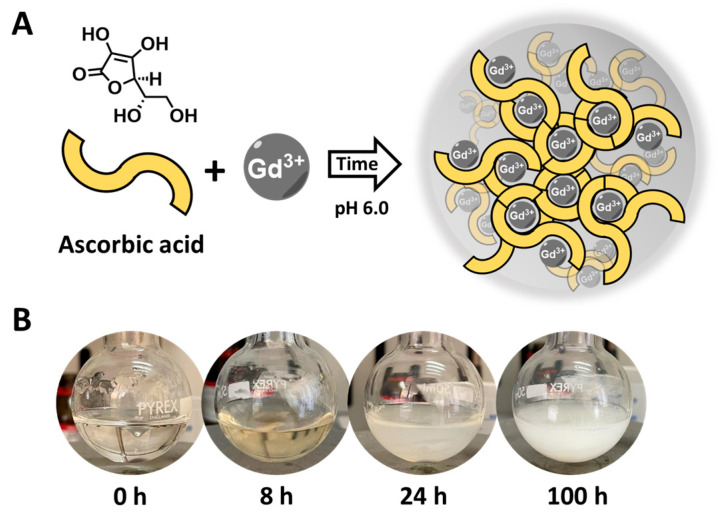
(**A**) General scheme of the reaction between Gd(III) chloride and ascorbic acid, leading to Gd-Asc nanoparticles; (**B**) Digital photos of the reaction mixture over time.

The synthesis of Gd-Asc nanoparticles was achieved using a similar approach described in the literature for comparable nanoparticles [36]. Briefly, a solution (15 mL) containing hydrated GdCl_3_ and ascorbic acid in a 1.00:2.66 molar ratio was prepared. The pH was maintained at 6.0 to promote the coordination of the ascorbic acid with the metal ions (Figure 1A). Initially, the reaction results in the creation of small units of Gd(III)-ascorbate molecular complexes. These complexes gradually develop into nanoparticles with varying aggregation states, as indicated by the increasing turbidity of the solution over time (Figure 1B). After 24 h, a stable white suspension is formed. However, with prolonged reaction times (e.g., 100 h), the suspension becomes unstable, resulting in the formation of large aggregates that quickly precipitate (Figure 1B).

**Figure 2:**
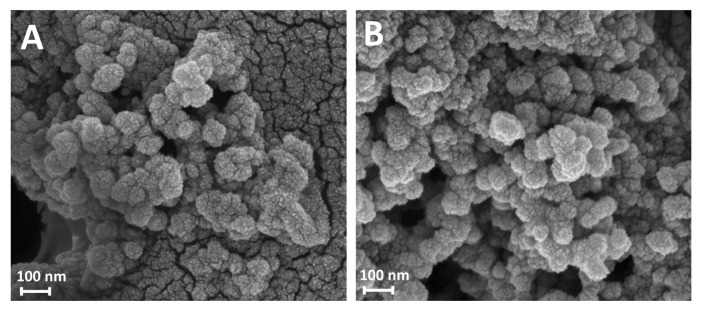
SEM micrographs of Gd-Asc-24 (**A**) and Gd-Asc-100 (**B**).

The morphology of the purified nanoparticles obtained after 24 and 100 h of reaction (Gd-Asc-24 and Gd-Asc-100, respectively) was examined using scanning electron microscopy (SEM). Micrographs collected for both samples revealed spheroidal nanoparticles with diameters of approximately 70 nm (Figure 2A,B), forming heterogeneous aggregates of varying sizes. These results indicate that the increased turbidity of the suspension from 24 to 100 h of reaction is not due to a growth in particle size, which remains comparable in both cases, but rather to a higher degree of aggregation in the aqueous solution.

**Figure 3:**
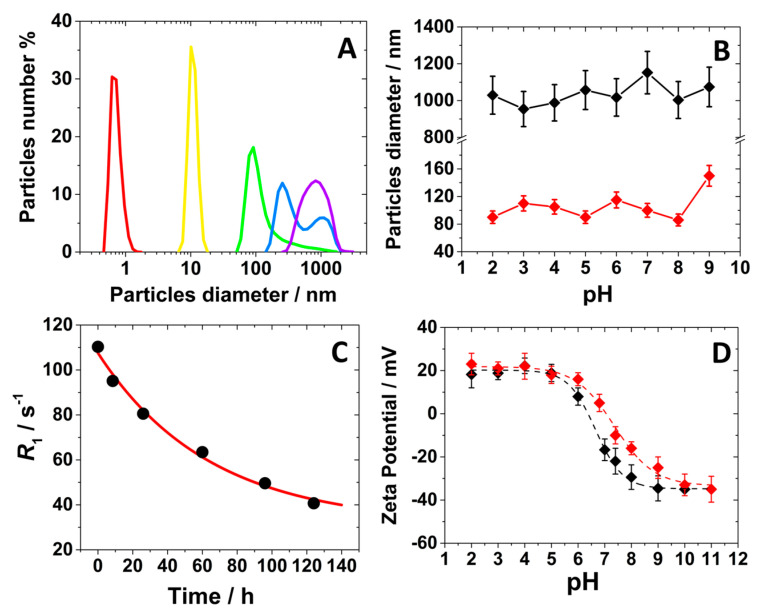
(**A**) DLS analysis for the reaction mixture at different times: **▪** = 0 h, **▪** = 8 h, **▪** = 24 h, **▪** = 31 h, **▪** = 100 h; (**B**) hydrodynamic diameter of the nanoparticles as a function of pH for Gd-Asc-100 (◆) and Gd-Asc-24 (◆); (**C**) longitudinal relaxation rate overt time for the reaction mixture at 32 MHz and 298 K; (**D**) Z-potential dependence on pH for Gd-Asc-100 (◆) and Gd-Asc-24 (◆).

This comment was supported by analyzing the particles size distribution in aqueous solution by Dynamic Light Scattering (DLS) analysis over time. At the early stage of the synthesis, the solution consists of molecular units smaller than 1 nm (Figure 3A). After 8 h, nanoparticles begin to form with hydrodynamic diameters around 10 nm, continuing to grow until reaching larger sizes at 24 h (ca. 80 nm in aqueous solution) (Figure 3A and Table 1). At extended reaction times, the particle size remains unchanged, as confirmed by SEM analysis, but the nanoparticles tend to assemble into larger aggregates ranging from 300 nm to 1 μm (Figure 3A and Table 1). Notably, the size of these nanoparticles remained unaffected by variations in the pH of the solution (Figure 3B).

The UV-Visible spectrum obtained from the aqueous suspension of Gd-Asc-24 revealed a prominent broad absorption band around 300 nm, which is approximately 50 nm higher than that observed for pure ascorbic acid in solution. This shift is attributed to the coordination of ascorbic acid with Gd(III) ions, influencing the π-π* transitions of the ascorbate units (Figure S1). The coordination of Gd(III) with ascorbate was also confirmed by IR spectroscopy of the nanoparticles dispersed in a KBr matrix. Notably, in the high wavenumber region, the distinct absorption bands of the pure ascorbate OH groups (3500–3200 cm^−1^) were replaced by a broad band centered around 3250 cm^−1^ following Gd(III) complexation, indicating coordination through hydroxyl functionalities (Figure S2) [36].

The formation of nanoparticles was also monitored by analyzing the longitudinal relaxation rate (*R*_1_) at 32 MHz and 298 K of the suspensions over time. Interestingly, an inverse relationship between the relaxation rate value and particle growth was observed. Indeed, the *R*_1_ of the suspension decreased monoexponentially over time, correlating with an increase in the hydrodynamic diameter (Figure 3C). This can be explained by the gradual internalization of Gd(III) ions within the coordination polymer framework with the increase of the particles size and aggregation state, leading to a progressive reduction in the number of Gd(III) ions exposed on the surface of the nanoparticles in interaction with water molecules. This process significantly affects the longitudinal relaxation rate mainly controlled by the dipolar nature of the Gd(III)-water relaxation mechanism, which follows a 1/*r*^6^ dependence (where *r* is the average distance between Gd(III) ions and the water protons) [1,37]. Consequently, only surface-exposed Gd(III) ions, which are in closer proximity to water molecules, effectively contribute to relaxation rate. Finally, Z-potential measurements were performed on the suspensions of the Gd-Asc-24 and Gd-Asc-100 nanoparticles, showing a positive charge of approximately +20 mV in acidic conditions (pH < 6.0), attributed to the presence on the surface of Gd(III) ions (Figure 3D and Table 1). At physiological pH, we observed a gradual shift toward a negative surface charge (Table 1), likely due to the deprotonation of ascorbate groups on the nanoparticle surface [38], with −40 mV observed for both systems in basic conditions (Figure 3D).

Gd-Asc-24 and Gd-Asc-100 suspensions differ also in both nanoparticles content and Gd(III) concentration. In the case of Gd-Asc-100, the amount in mg of solid sample was approximately six times higher than that in Gd-Asc-24 (Table 1). Additionally, the Gd(III) concentration, as determined by ICP-MS analysis, was 8.2 mM, about five times higher than that measured for Gd-Asc-24 (Table 1). These findings support the hypothesis of increased nanoparticle aggregation in the suspension obtained after 100 h of reaction.

**Figure 4:**
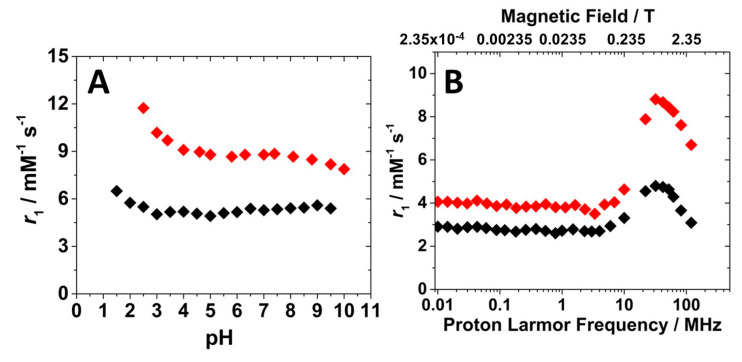
(**A**) Relaxivity dependence on pH (**A**) and ^1^H NMRD profiles at 298 K and pH 7.4 (**B**) for Gd-Asc-100 (◆) and Gd-Asc-24 (◆).

The relaxometric properties of the Gd-Asc-24 and Gd-Asc-100 suspensions were investigated in detail. In an early stage, the relaxivity dependence on pH was recorded at 32 MHz and 298 K, providing valuable information about the stability of the suspensions (Figure 4A). Relaxivity (*r*_1_) is defined as the longitudinal relaxation rate of water protons per 1 mM concentration of Gd(III). Gd-Asc-24 suspension showed higher relaxivity values across the entire pH range examined, in respect to Gd-Asc-100. At neutral pH, the *r*_1_ value for Gd-Asc-24 was approximately 9 mM^−1^ s^−1^ at 32 MHz and 298 K, +30% in respect to Gd-Asc-100, and ca. two times greater than that of commercially available MRI probes (Figure 4A) [3]. For both suspensions, an increase in *r*_1_ was observed at acidic pH values below 4, likely due to a gradual increase in the hydration state of Gd(III) ions. Within the pH range from 4 to 9, the *r*_1_ value remains stable, indicating good chemical stability of the nanoparticles (Figure 4A). Minor fluctuations in *r*_1_, observed at pH > 9 for Gd-Asc-24, may be tentatively attributed to slight structural changes in the nanoparticles. The *r*_1_ values were also measured as a function of the applied magnetic field, thus obtaining the Nuclear Magnetic Relaxation Dispersion (NMRD) profiles. These profiles provide valuable insights into the relaxation mechanisms of the system. The ^1^H NMRD profiles for both suspensions are shown in Figure 4B.

The shape of ^1^H NMRD profiles was similar for both suspensions. At low magnetic fields (<10 MHz), *r*_1_ values remained constant. In contrast, at higher Larmor frequencies, a bell-shaped peak appeared, centered around 30–40 MHz. This behavior is characteristic of hydrated Gd(III)-based systems with restricted mobility in the solution. Notably, Gd-Asc-100 exhibited lower *r*_1_ values, in particular at clinical magnetic fields (1.5–3.0 T), if compared to Gd-Asc-24 suspension (Figure 4B). This behavior can be explained by the different degrees of aggregation between the two formulations. In the presence of large aggregates, a smaller fraction of Gd(III) ions are exposed on the surface and directly interact with water molecules, compared to the total amount of metal contained within the particles. A substantial portion of Gd(III) ions, primarily located in the core of the aggregates, although included in the *r*_1_ calculation, are silent by the relaxometric point of view, as has been observed in other paramagnetic nanosystems [33,39]. For this reason, given the high complexity of these nanosystems and the limited control over the hydration state of surface Gd(III) ions, a quantitative analysis of these profiles, using dedicated mathematical models [40,41,42,43,44], would be highly speculative and potentially inappropriate. However, several conclusions can be qualitatively proposed: (i) the *r*_1_ values for both nanoparticles at high magnetic fields indicate that, on average, the Gd(III) ions are hydrated; (ii) both nanoparticles exhibit a slow tumbling motion in solution, as evidenced by the broad peak at high magnetic fields in the NMRD profiles; and (iii) the water exchange rate is sufficiently rapid not to limit the relaxivity, as demonstrated by the analysis of *r*_1_ as a function of temperature at 32 MHz (Figure S3). A gradual decrease in the *r*_1_ value with increasing temperature from 283 to 323 K is characteristic of paramagnetic probes with an intermediate-fast water exchange regime.

### 2.2. Gd(III)/Eu(III)-Ascorbate NPs

Given the capability of these nanoparticles to internalize in the structure Gd(III) ions, a possible interesting approach to confer additional properties relies on the doping of the NPs with other lanthanides ions, such as Eu(III), in order to impart also luminescent features, obtaining a multimodal imaging probe. This idea was then followed by simply using an Ln(III) source during the coordination polymer formation, a mixture of Gd(III) and Eu(III) chlorides species. The attention was mainly focused on the systems obtained after 24 h of reaction. In order to assess how the doping of the material impacts the NPs properties, two different formulations were synthesized and characterized, either being used for the reaction a 90/10 and a 70/30 Gd(III)/Eu(III) molar ratio (Gd/Eu-Asc_1 and Gd/Eu-Asc_2, respectively).

These NPs were found to be reasonably morphologically comparable with the previously described nanoparticles, with a NP dimension below 80 nm (Figure S4). In addition, the UV-Visible spectra of the two formulations showed the same profile observed for the parent Gd-Asc-24 sample (Figure S1). ICP-MS analysis was performed to determine the Ln(III) content, revealing a stoichiometry that closely matches the molar ratio used in the synthetic procedure (Table 2). This suggests a non-selective incorporation of the Ln(III) ions during particle formation. The ICP-MS results were further supported by EDX measurements (Figure S5, Tables S1 and S2). The concentration of the Ln(III) ions in an aqueous solution for the different formulations are reported in Table 2.

**Figure 5:**
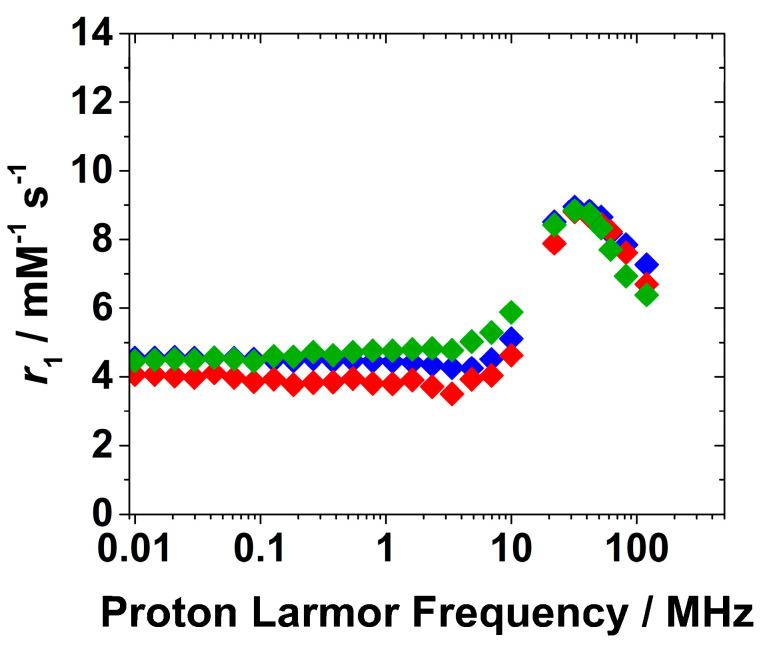
^1^H NMRD profiles of Gd-Asc-24 (◆), Gd/Eu-Asc_1 (◆) and Gd/Eu-Asc_2 (◆) at pH 7.0 and 298 K.

The two different suspensions were then characterized by ^1^H NMR relaxometry, and the ^1^H NMRD profiles at 298 K and pH 7.0 were recorded and reported in Figure 5.

Interestingly, the ^1^H NMRD profiles for both formulations show no significant differences at high magnetic fields if compared to Gd-Asc-24 NPs, indicating that Eu(III) doping does not substantially affect the *r*_1_ values and the NMRD shape. The minor differences observed at low fields, below 10 MHz in the ^1^H NMRD profiles of nanoparticles containing Eu(III) may instead result from small changes in the electronic properties of nearby Gd(III) centers. This hypothesis could be plausible considering that *r*_1_ values at low magnetic fields are highly sensitive to electron relaxation properties of the probe.

The luminescent characteristics of aqueous suspensions containing these nanoparticles were studied. When exposed to UV light at 365 nm, the Eu(III)-doped nanoparticles distinctly differed from the Gd-Asc-24. Whereas the original system emits a bright white light, the Eu(III)-doped nanosystems emit a red light, especially notable in the Gd/Eu-Asc_2 formulation.

The excitation spectra of the aqueous suspensions of Gd/Eu-Asc_1 and Gd/Eu-Asc_2 were recorded by monitoring the Eu^3+^ emission at 592 nm. Both spectra exhibited the characteristic intra-4f^6^ electronic transitions of Eu^3+^, with a prominent peak at ca. 395 nm corresponding to the ^7^F_0_ → ^5^L_6_ transition. Additional bands observed at 275 and 315 nm are attributed to the ^8^S_7/2_ → ^6^I*_J_* and ^8^S_7/2_ → ^6^P*_J_* transitions of Gd^3+^, respectively (Figure S6) [45]. The presence of these bands suggests an energy transfer process from Gd^3+^ to Eu^3+^, likely facilitated by the partial overlap of their energy levels, as illustrated in the corresponding Jablonski diagram. In the case of Gd/Eu-Asc_2, an additional asymmetric band centered at 255 nm is observed, which is attributed to a charge transfer transition (O^2−^ → Eu^3+^). This feature becomes particularly prominent at higher Eu^3+^ concentrations (Figure S6) [46].

**Figure 6:**
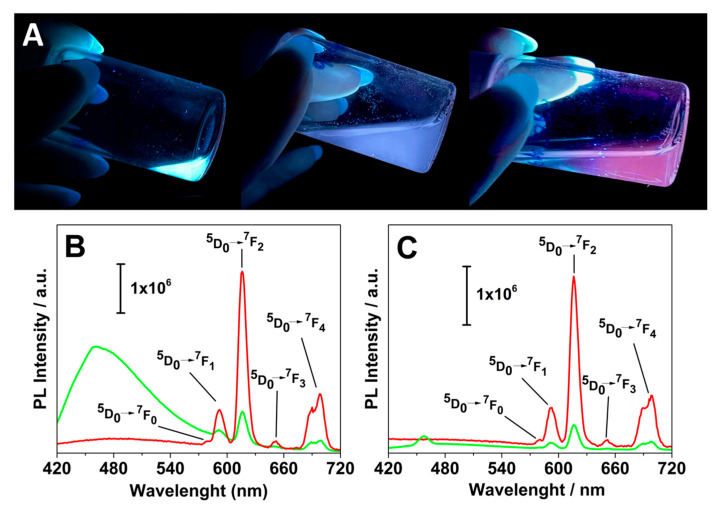
(**A**) Digital photos of the different suspensions upon irradiation with an UV torchlight: from left to right, Gd-Asc-24, Gd/Eu-Asc_1 and Gd/Eu-Asc_2 (pH 7.0, 298 K); Photoluminescence spectra of Gd/Eu-Asc_1 (**B**) and Gd/Eu-Asc_2 suspensions (**C**) under excitation at 280 nm (**▪**) and 395 nm (**▪**).

Emission spectra recorded by photoluminescence spectroscopy provided further insight into the radiative processes involved. For Eu(III)-doped nanosystems, two different PL spectra were recorded upon excitation at 280 (absorption ascribed to the ^8^S_7/2_−^6^I_J_ main transition of Gd(III)) and 395 nm (absorption associated with ^7^F_0_−^5^L_6_ electron transition of Eu(III)) [47,48]. Both nanoparticles exhibit the characteristic emission of the intra-electronic levels of Eu(III) (^5^D_0_ → ^7^F*_J_*, with *J* = 0–4), for both excitation conditions (λ_exc_ of 280 and 395 nm). Notably, direct excitation of Gd(III) at 280 nm leads to a pronounced enhancement of the Eu(III) emission intensities in both formulations in the 550–720 nm range (Figure 6B,C). This indicates a substantial energy transfer from Gd(III) to Eu(III), consistent with observations reported for other bifunctional inorganic nanoparticles [45,49].

The intensity ratio of the bands at 615 nm (assigned to the electric dipole ^5^D_0_ → ^7^F_2_) and 592 nm (the magnetic dipole ^5^D_0_ → ^7^F_1_ transition) [47] defines the asymmetry factor R parameter that provides valuable insight into the symmetry of the coordination environment around Eu(III) centers [50,51,52]. The R factor approaches 0 for highly symmetric Eu(III) sites, whereas higher values indicate a lower symmetry environment. For these nanoparticles, the calculated R values are found to be between 2 and 4 (Table S3) under excitation at 280 and 395 nm, suggesting a highly asymmetric coordination environment around the Eu(III) centers. This is reasonable considering the uncontrolled architecture of the coordination polymer framework.

An emission peak at 480 nm is observed in the spectra upon excitation at 395 nm, most prominently in the Gd/Eu-Asc_1 formulation. This emission aligns well with previously reported data and may be associated with light-emitting structural defects [36,53]. This interpretation is further supported by the presence of the same emission band in the parent Gd-Asc-24 under excitation at both 280 and 395 nm (Figure S7). However, a solid assessment of this emission is particularly challenging. Previous studies have qualitatively examined this aspect as well. Basu et al. suggest that this band may partly arise from the luminescence of ascorbic acid due to its π-conjugated groups and heteroatoms [53]. However, this component significantly intensified during the formation and enlargement of the ascorbate nanoparticles, even without the presence of Gd(III) or Eu(III) ions [53]. Consequently, this emission is linked to the nanoparticle structure, and minor variations in their architecture noticeably affect the emission intensity.

## 3. Materials and Methods

### 3.1. Synthetic Procedures

*Chemicals and Materials*. All chemicals were purchased from Sigma-Aldrich Co. (St. Louis, MO, USA) and used without further purification.

*Synthesis of the Gd(III)-Ascorbate Nanoparticles.* The synthesis was carried out adapting a procedure already described in the literature [36]. Fifty mg of GdCl_3_·6H_2_O were firstly dissolved in 15 mL of milliQ water (Millipore, Burlington, MA, USA, 0.135 mmol, 9 mM solution). Then, 63 mg of ascorbic acid were slowly added (0.36 mmol, 24 mM). The pH was raised and maintained to 6.0 by adding NaOH 1M in order to promote the formation of the nanoparticles. At 8 h, the solution turns slightly yellow, while at 24 h a yellow-white suspension is clearly visible. The suspension progressively increases turbidity for longer times (Figure 1B). In order to characterize the nanoparticles at different reaction times, two different reaction mixtures were dialyzed using a membrane with cut-off of 1000 Da against an aqueous solution at pH 6.0, after 24 (Gd-Asc-24) and 100 h (Gd-Asc-100) from the beginning of the synthesis. All suspensions were kept under a nitrogen atmosphere to prevent oxidation of ascorbic acid.

*Synthesis of the Gd(III)/Eu(III)-Ascorbate Nanoparticles.* The synthesis was carried out following the previously described procedure but using as metal ion source a mixture of GdCl_3_·6H_2_O and Eu(NO_3_)_3_·5H_2_O. Different molar ratios of Gd(III):Eu(III) were explored for the formation of the samples, namely Gd/Eu-Asc_1 with 90:10 molar ratio (8.1 mM of Gd(III) and 0.9 mM of Eu(III)) and Gd/Eu-Asc_2 with 70:30 molar ration (6.3 mM of Gd(III) and 2.7 mM of Eu(III)). The suspensions were then dialyzed as reported for the Gd(III)-ascorbate nanoparticles and were kept under a nitrogen atmosphere to prevent oxidation of ascorbic acid.

### 3.2. Characterization Techniques

*DLS characterization.* Dynamic light scattering (DLS) and Z-potential experiments were carried out at room temperature by using a Malvern Zetasizer NanoZS (Malvern, UK) operating in a particle size range from 0.6 nm to 6 mm and equipped with a He-Ne laser with λ = 633 nm.

*SEM measurements*. Scanning electron microscopy measurements were performed using a ZEISS GeminiSEM 360 (Jena, Germany), a high-resolution field emission scanning electron microscope. After deposition of 1 mg of sample on the sample holder, NPs were coated with a few-nanometer-thick platinum layer using an Emitech K575X metallizer (Laughton, UK), to enhance conductivity and minimize charging effects during the analysis. Elemental composition was assessed by energy-dispersive X-ray spectroscopy (EDX), using an Ultim Max 65 detector (Oxford Instruments, Abingdon, UK) coupled to the SEM. A 10 keV accelerating voltage was applied, and spectra were acquired in both point and area scan modes. Elemental analysis was performed qualitatively using AZtec software (Oxford Instruments, Abingdon, UK).

*ICP-MS*. The determination of Gd(III) and Eu(III) content in the different samples was carried out using a Thermo Scientific (Waltham, MA, USA) iCAP™ RQ inductively coupled plasma mass spectrometer with single quadrupole technology. The system includes an ESI (Omaha, NE, USA) PFA 100 MicroFlow nebulizer, a Peltier-cooled quartz spray chamber operating at 3 °C, a 2.0 mm ID quartz injector, and a demountable quartz torch. Uptake from the samples was managed using an ESI (Omaha, USA) SC-4 DX autosampler system. To overcome spectral interferences, the Collision Cell Technology (CCT) was used with He gas at 3.5 mL/min and a kinetic energy discrimination (KED) barrier of 2 V. Calibration curves were obtained by diluting a multielement standard solution. The samples were firstly digested in 65% HNO_3_ and then diluted with 1% HNO_3_ to the desired concentrations prior to the analysis.

*UV−Vis analysis.* UV-Visible spectra were recorded at RT in the range 400−700 nm with a resolution of 1 nm, using a double-beam PerkinElmer Lambda 900 spectrophotometer (Waltham, MA, USA).

*IR spectroscopy.* Infrared (IR) spectra were collected in the range 4000–400 cm^−1^ with a Bruker Equinox 55 spectrometer (Billerica, MA, USA): all measures were collected by diluting the samples in KBr matrix.

*Relaxometric characterization.* 1/*T*_1_ ^1^H Nuclear Magnetic Relaxation Dispersion (NMRD) profiles were measured on a Fast-Field Cycling (FFC) Stelar SmarTracer Relaxometer (Stelar, Mede, Italy) over magnetic field strengths ranging from 0.00024 to 0.25 T (0.01–10 MHz proton Larmor Frequencies). The relaxometer operates under computer control with an absolute uncertainty in 1/*T*_1_ of ±1%. Data in the 20–120 MHz frequency range were collected with a High Field Relaxometer (Stelar) equipped with the HTS-110 3T Metrology Cryogen-free Superconducting Magnet (Mede, Italy). The temperature was controlled during the measurements with a Stelar VTC-91 airflow heater (Mede, Italy) equipped with a copper-constantan thermocouple (uncertainty of ±0.1% °C). The real temperature inside the probe head at the sample position was monitored by a Fluke 52k/j digital thermometer (Fluke, Zürich, Switzerland). Longitudinal relaxation rates were collected using the standard inversion recovery sequence (sixteen experiments, three scans) with a typical 90° pulse width of 3.5 μs by vortexing the suspension prior measurements, and the reproducibility of the data was within ±1.0%. The relaxivity (*r*_1_, mM^−1^ s^−1^) at different magnetic fields was obtained by measuring the longitudinal relaxation rates of the paramagnetic suspensions and subtracting the diamagnetic contribution of the pure water; the final values were then divided by the mM concentration of the paramagnetic ion in the suspension. In the case of the Eu(III)-doped nanoparticles, the relaxivity of the systems was calculated taking into account the Gd(III) concentration only, given the negligible contribution of Eu(III) to longitudinal relaxation rate in the explored experimental conditions. The pH dependence was measured by changing the pH of the sample by addiction of 0.1 M NaOH or 0.1 M HCl.

*Luminescence measurements.* Excitation and luminescence spectra for the different suspensions were measured with a Horiba Jobin Yvon Model IBH FL-332 Fluorolog 3 spectrometer (Kyoto, Japan), at different excitation (395 and 280 nm). Digital photos of the suspensions were obtained by irradiating samples with an UV torch light (Alonefire^®^, Shenzhen, China, 365 nm, 10 W).

## 4. Conclusions

In this work, the magnetic and morphological properties of Gd(III)-based ascorbate nanoparticles were investigated, with particular attention given to evaluating how synthesis time affects their final characteristics. The results indicate that particles obtained after 24 h of reaction exhibited the most favorable properties, including (1) particle sizes below 80 nm; (2) good suspension stability across varying pH conditions; and (3) relaxivity values at clinical magnetic fields exceeding those typically observed for low-molecular-weight complexes used in clinical settings.

Additionally, the possibility of incorporating both Gd(III) and Eu(III) within the same particles at varying molar ratios was explored. The introduction of luminescent centers did not compromise the magnetic properties of the nanoparticles, while imparting optical properties that can be activated either by direct excitation of Eu(III) or via energy transfer from Gd(III) to Eu(III). A significant enhancement in luminescence intensity was observed upon excitation of Gd(III).

In light of these promising results, these nanoparticles represent a solid platform for the development of mono- and bimodal probes for diagnostic applications.

## Figures and Tables

**Table 1:** Chemical and morphological properties of the two Gd-Asc formulations in aqueous solution.

	Gd-Asc-24	Gd-Asc-100
Amount of solid (mg/mL)	1.0 ± 0.1	6.2 ± 0.1
Gd(III) concentration (mM)	1.6 ± 0.1	8.2 ± 0.3
Hydrodynamic diameter (nm)	80 ± 7	955 ± 103
Z-potential at pH 7.4 (mV)	−10 ± 4	−20 ± 6

**Table 2:** Gd(III) and Eu(III) concentration in the Gd/Eu-Asc_1 and Gd/Eu-Asc_2, determined by ICP-MS.

	Gd/Eu-Asc_1	Gd/Eu-Asc_2
[Gd(III)]/mM	1.60 ± 0.10	0.75 ± 0.06
[Eu(III)]/mM	0.20 ± 0.03	0.38 ± 0.04

## Data Availability

The datasets supporting this article have been uploaded as part of the ESI. Additional data can be provided upon reasonable request from the authors.

## Supplementary Materials

The following supporting information can be downloaded at: https://www.mdpi.com/article/10.3390/molecules30132689/s1. Figure S1. UV-Visible spectra of ascorbic acid (black), Gd-Asc-24 (red), Gd/Eu-Asc_1 (blue) and Gd/Eu-Asc_2 (green); Figure S2. IR spectra of ascorbic acid (red) and Gd-Asc-24 (black) in KBr matrix; Figure S3. Relaxivity dependence on temperature recorded for Gd-Asc-24 (red) and Gd-Asc-100 (black) at 32 MHz; Figure S4. SEM micrographs of Gd/Eu-Asc_1 (A) and Gd/Eu-Asc_2 (B); Figure S5. EDX spectra recorded for Gd/Eu-Asc_1 (A) and Gd/Eu-Asc_2 (B); Table S1. Gd(III) and Eu(III) content in Gd/Eu-Asc_1 obtained from EDX analysis; Table S2. Gd(III) and Eu(III) content in Gd/Eu-Asc_2 obtained from EDX analysis; Figure S6. Excitation spectra for Gd/Eu-Asc_1 (blue) and Gd/Eu-Asc_2 (green) by monitoring the emission peak at 592 nm; Figure S7. Photoluminescence spectra recorded for Gd-Asc-24 upon excitation at 280 (red) and 395 nm (green); Table S3. R asymmetry factor obtained for Gd/Eu-Asc_1 and Gd/Eu-Asc_2 at different excitation wavelengths.
