# Supplementary material for: Paramagnetic and Luminescent Properties of Gd(III)/Eu(III) Ascorbate Coordination Polymers
Source: Molecules. 2025 Jun 21;30(13):2689. doi: 10.3390/molecules30132689 (PMC12251071; doi:10.3390/molecules30132689)
Supplement: Supplementary file 1 [file molecules-30-02689-s001.zip › molecules-3699739-supplementary.pdf]

Paramagnetic and luminescent properties of Gd(III)/Eu(III) Ascorbate Coordination Polymers

Marco Ricci[1] and Fabio Carniato[1,*]

[1]Dipartimento di Scienze e Innovazione Tecnologica, Università del Piemonte Orientale "A. Avogadro", Viale T. Michel 11, 15121 Alessandria, Italy.

Correspondence: E-mail: fabio.carniato@uniupo.it, Tel.: +390131360217

Table of contents:

Figure S1. UV-Visible spectra of ascorbic acid (black), Gd-Asc-24 (red), Gd/Eu-Asc_1 (blue) and Gd/Eu-Asc_2 (green). 1

Figure S2. IR spectra of ascorbic acid (red) and Gd-Asc-24 (black) in KBr matrix. 1

Figure S3. Relaxivity dependence on temperature recorded for Gd-Asc-24 (red) and Gd-Asc-100 (black) at 32 MHz. 2

Figure S4. SEM micrographs of Gd/Eu-Asc_1 (A) and Gd/Eu-Asc_2 (B). 2

Figure S5. EDX spectra recorded for Gd/Eu-Asc_1 (A) and Gd/Eu-Asc_2 (B). 3

Table S1. Gd(III) and Eu(III) content in Gd/Eu-Asc_1 obtained from EDX analysis. 3

Table S2. Gd(III) and Eu(III) content in Gd/Eu-Asc_2 obtained from EDX analysis. 3

Figure S6. Excitation spectra for Gd/Eu-Asc_1 (blue) and Gd/Eu-Asc_2 (green) by monitoring the emission peak at 592 nm. 4

Figure S7. Photoluminescence spectra recorded for Gd-Asc-24 upon excitation at 280 (red) and 395 nm (green). 4

Table S3. R asymmetry factor obtained for Gd/Eu-Asc_1 and Gd/Eu-Asc_2 at different excitation wavelengths. 5

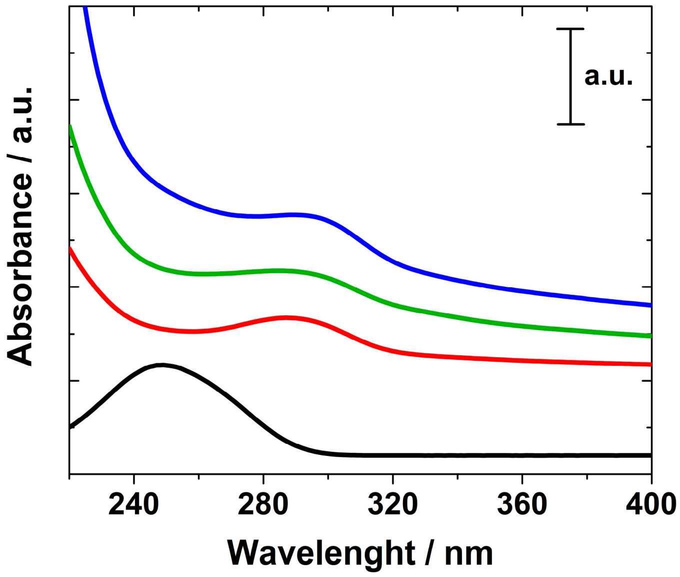

Figure S1. UV-Visible spectra of ascorbic acid (black), Gd-Asc-24 (red), Gd/Eu-Asc_1 (blue) and Gd/Eu-Asc_2 (green).

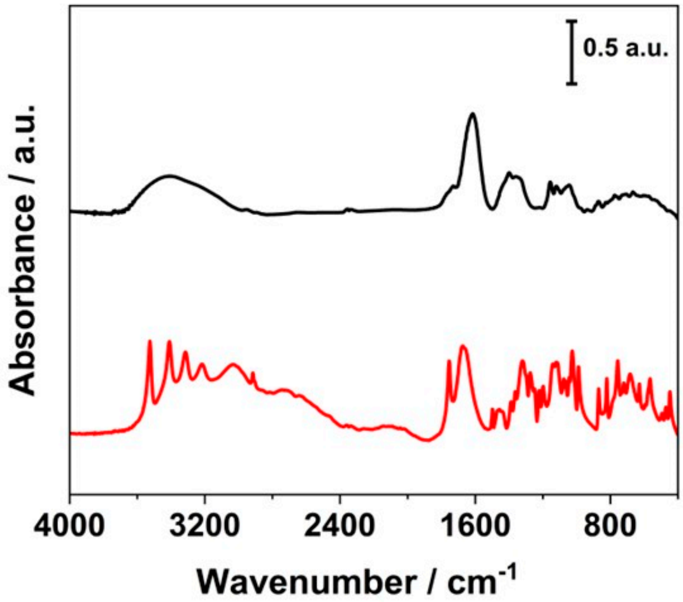

Figure S2. IR spectra of ascorbic acid (red) and Gd-Asc-24 (black) in KBr matrix.

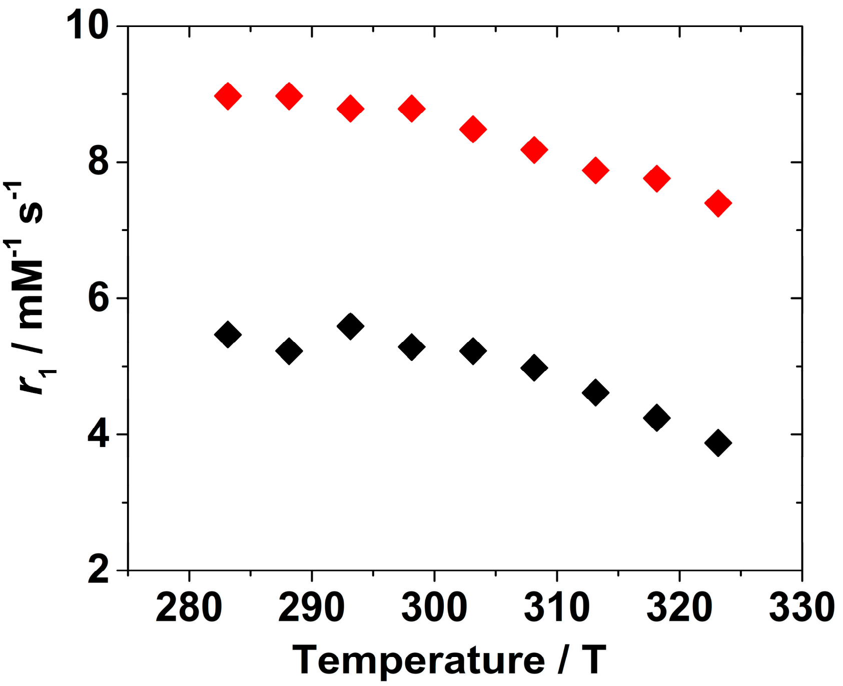

Figure S3. Relaxivity dependence on temperature recorded for Gd-Asc-24 (red) and Gd-Asc-100 (black) at 32 MHz.

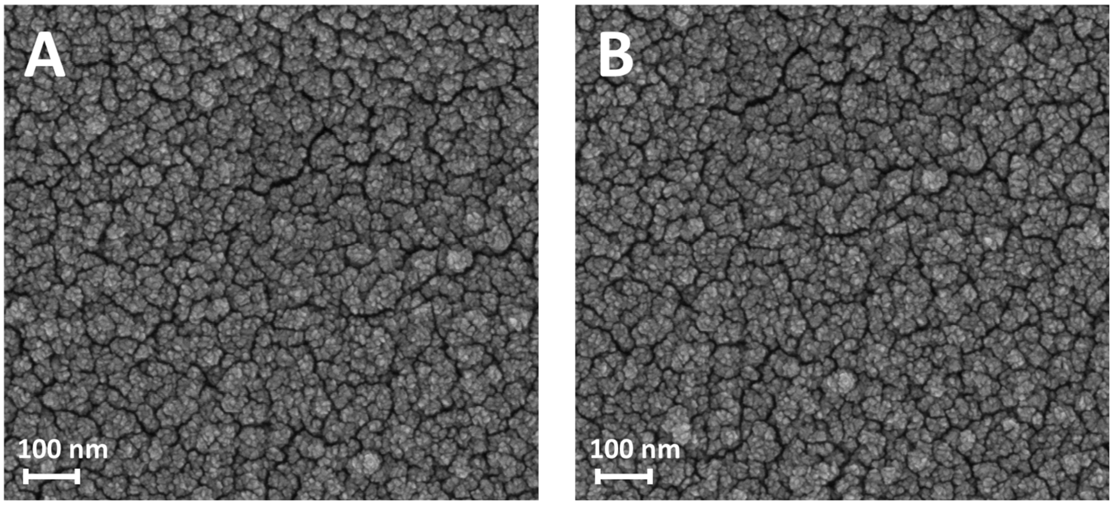

Figure S4. SEM micrographs of Gd/Eu-Asc_1 (A) and Gd/Eu-Asc_2 (B).

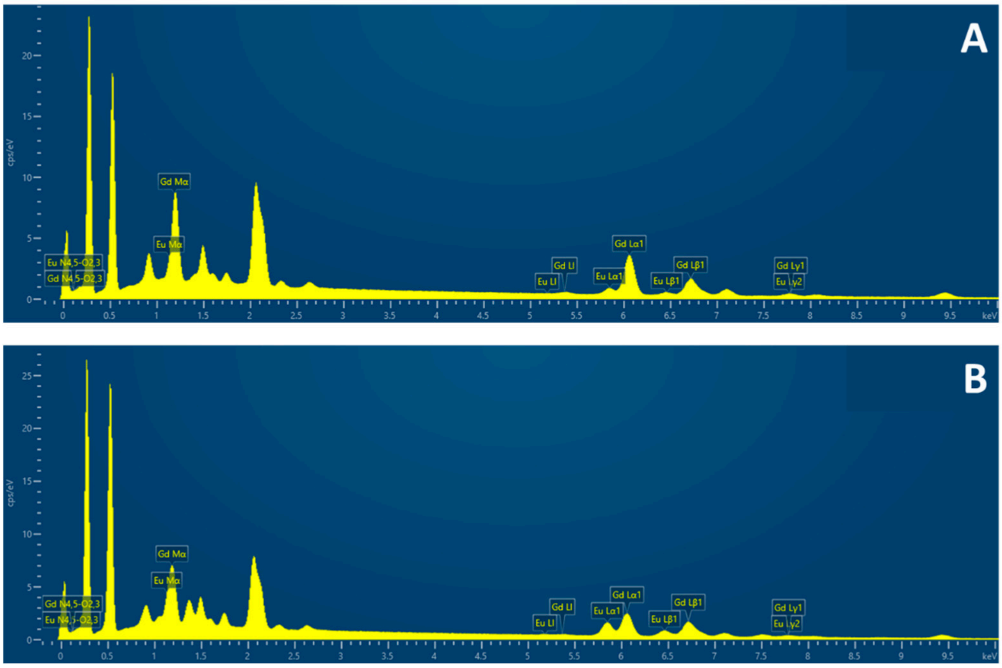

Figure S5. EDX spectra recorded for Gd/Eu-Asc_1 (A) and Gd/Eu-Asc_2 (B).

Table S1. Gd(III) and Eu(III) content in Gd/Eu-Asc_1 obtained from EDX analysis.

Element	Wt %	Atomic %
**Eu**	12.00	12.37
**Gd**	88.00	87.63
**Total**	100.00	100.00

Table S2. Gd(III) and Eu(III) content in Gd/Eu-Asc_2 obtained from EDX analysis.

Element	Wt%	Atomic %
**Eu**	34.41	35.19
**Gd**	65.59	64.81
**Total**	100.00	100.00

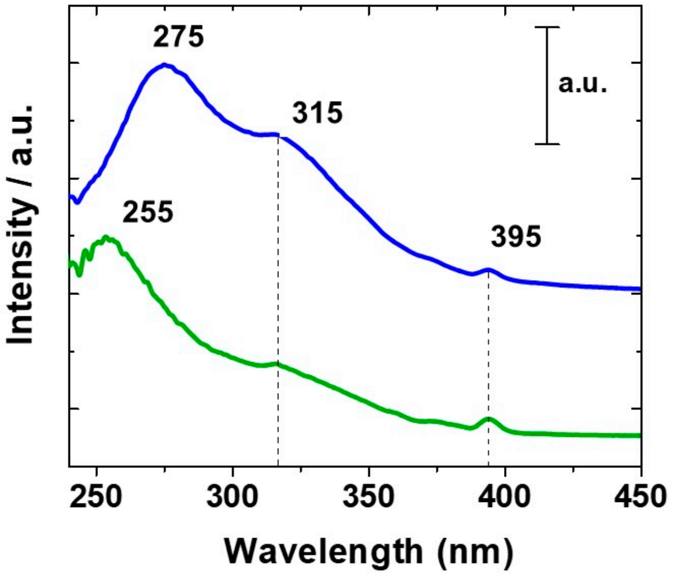

Figure S6. Excitation spectra for Gd/Eu-Asc_1 (blue) and Gd/Eu-Asc_2 (green) by monitoring the emission peak at 592 nm.

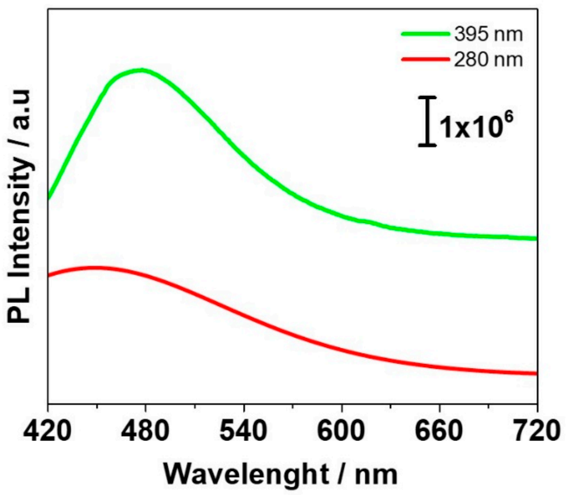

Figure S7. Photoluminescence spectra recorded for Gd-Asc-24 upon excitation at 280 (red) and 395 nm (green).

Table S3. R asymmetry factor obtained for Gd/Eu-Asc_1 and Gd/Eu-Asc_2 at different excitation wavelengths.

Excitation (λ)	Gd/Eu-Asc_1	Gd/Eu-Asc_2
280 nm	1.8	4.2
395 nm	3.4	4.1
